# Compression Garments for Medical Therapy and Sports

**DOI:** 10.3390/polym10060663

**Published:** 2018-06-14

**Authors:** Ying Xiong, Xiaoming Tao

**Affiliations:** 1 Institute of Textiles and Clothing, The Hong Kong Polytechnic University, Hung Hom, Hong Kong, China; ying.xy.xiong@polyu.edu.hk; 2 Department of Biomedical Engineering, The Hong Kong Polytechnic University, Hung Hom, Hong Kong, China

**Keywords:** compression garment, pressure therapy, sportswear, material, knitting, design, modeling

## Abstract

Compression garments are elastic clothing with an engineered compression gradient that can be worn on limbs, upper, lower, or full body to use for therapy and sports. This article presents an overview and review on the compression garments and concentrates on the design of compression garments with an appropriate pressure for specific applications. It covers the types of compression garments, fibers and yarns, knitted fabric construction, garment design, an evaluation system, and pressure measurement and modeling. The material properties, fabric properties, pressure modeling, and the garment design system presents the prediction, design, and fabrication of the compression garments. Lastly, the research status and directions are discussed.

## 1. Introduction

Compression garments are special clothing containing elastomeric fibers and yarns used to apply substantial mechanical pressure on the surface of needed body zones for stabilizing, compressing, and supporting underlying tissues [1]. They have been widely researched and utilized in the fields of medical applications, athletic applications, and body-shaping applications [2]. 

The first mention of compression therapy appeared in the Corpus Hippocraticum (450–350 BC) [3]. It was believed that pressure exerted could levitate the side effects of gravity and uphold posture in order to benefit wound healing of the lower limbs. Compression therapy, which is applied with bandages, started out as a form of conservative treatment for varicose veins in 1440. However, the commercial adoption of bandages did not appear until the end of the 19th century [4]. The first modern elastic compression stockings with gradual compression were also fabricated in the 19th century in England. Compression therapy has also been used for the last 50 years for burn care and has been accepted to help minimize the formation of hypertrophic scars and enhance the maturation process of scars [5].

Compression garments have been utilized for medical reasons for many years. The use of compression garments has become widely common in sportswear. Sportswear with moderate compression distribution is widely used in athletics and fitness activities and is expected to enhance the performance of the athletes, decrease the possibility of injury, and accelerate the process of recovery [6,7,8]. Likewise, compression garments with a slight pressure designed to be tight fitting for body shaping purpose are becoming more popular.

The effectiveness, safety, pressure distribution, and retention of the compression garments are important aspects that have significant effects on the health of users. Among compression garments, the pressure performance including magnitude and durability is the key indicator, which is determined primarily by mechanical properties of the garment and garment fit [9]. Pressure exerted by the compression garment is one of the most important properties for evaluating therapy efficacy, comfort, health, and security. Denton [10] identified the pressure threshold of discomfort to be around 5.88–9.80 kPa (44.1–73.5 mmHg) depending on the individual subject and the part of the body concerned, which was greater but close to the average capillary blood pressure of 4.30 kPa (32.3 mmHg) near the skin surface. The pressure comfort zone for the normal condition is 1.96–3.92 kPa (14.7–29.4 mmHg), but it also depends on the individual condition of the treated body part and body position. An improper compression garment would influence the energy, work efficiency, and health of the wearer. Insufficient pressure will limit efficacy while too high of a pressure will make people feel uncomfortable, cause numbness to the body part, or even cause breathing difficulty and other serious damage to health [11]. Apart from pressure performance, the physical characteristics like air permeability, heat, moisture transmission, and tactile characteristics all have an influence on the comfort of compression garments. For medical compression garments, the antibacterial properties may be also taken into consideration.

This article presents an overview and review on the compression garments by concentrating on the different types of functional compression garments and the design of compression garments with appropriate pressure for specific applications. The information was collected by reviewing literature and searching electronic databases including PubMed, the Web of Science, and Google scholar. The systematic keywords include compression/pressure garment, compression/pressure therapy, chronic venous disease, hypertrophic scar, orthopedic support, sportswear, tight fitting garment, compression garment design, smart compression garment, elastomeric fiber/yarn/knitting fabric, pressure sensor, and pressure modeling. Since the authors major in materials and textile and have research experience on the compression stockings, the review mainly covers fibers and yarns, knitted fabric construction, garment design, an evaluation system, and the pressure measurement and modeling. The material properties, fabric properties, pressure modeling, and garment design system presents an instruction on the prediction, design, and fabrication of the compression garments. Lastly, the research status and directions are discussed.

## 2. Functional Compression Garments

Compression garments are individually designed and manufactured for a particular part of the body, stockings, bandages, sleeves, gloves, body suits, and face masks [2]. They have been utilized in the areas of chronic venous disease management, scar management, orthopedic supports, sportswear, and body shaping. 

### 2.1. Chronic Venous Disease and Edema Management

#### 2.1.1. Action Principle

The chronic venous disorder (CVD) is the most prevalent venous disorder in the venous system of the lower extremities and affects the human health in the world ranging from 5% to 30% in the adult population [12]. It is more prevailing with increasing age and it shows a sex difference with more in common with females than in males [13]. The typical manifestations of CVD include reticular veins, varicose veins, edema, pigmentation, eczema, lipodermatosclerosis, and healed and active venous ulcer [14]. The risk factors of CVD found to be linked with age, sex, pregnancy, obesity, heredity, phlebitis, and previous leg injury [15]. The environmental or behavioral factors are also associated with CVD like a sedentary life style, prolonged standing, inactivity, and occupation [16]. 

Two of the most universally accepted pathophysiology of venous insufficiency are primary valvular incompetence and congenital vein wall weakness [17,18,19]. Compression therapy is the conservative treatment and the footstone for the patients with venous insufficiency. The mechanisms of the operation are to increase venous flow velocity, reduce venous wall distension, and improve valvular function in order to reduce the venous hypertension of the limbs, improve venous hemodynamics, decrease the symptoms of the swollen extremity, and maintain the gradient pressure of the suffered leg. This ultimately helps improve the venous return from the distal to the proximal region [3]. 

Medical compression stockings (MCSs) or bandages with gradual compression from distal to proximal regions are usually utilized to conduct the compression therapy. There are two main principles of compression therapy. The first one is to create an enclosed system in order to allow an evenly distributed internal pressure in the leg. This principle involves the application of Pascal’s Law, which entails muscle movement generating a pressure wave that is distributed evenly in lower limbs during active and passive exercise. The compressive effect can reduce the diameter of veins by positioning valves and forcing the venous blood to return to the heart [20]. The second principle involves the application of Laplace’s Law in order to create a varied interface pressure based on limb shape as well as the tension of the stocking or bandage applied [21].

For lymphedema in the lower limb, the action principle of compression stockings is speculated to improve the calf muscle pumping on the veins as well as to help lymph propulsion by enhancing the extrinsic force such as contractions of the skeletal muscles adjacent to the lymphatic vessels [22]. With regard to the sitting position without leg exercises, wearing compression stockings plays a limited role in improving the extrinsic force. Another possible potential benefit of wearing compression stockings is to decrease capillary filtration [23]. 

#### 2.1.2. Application of Compression Stockings

Compression stockings come in different lengths including knee-high, low-thigh, high-thigh, and panty hose as well as multiple compressive magnitudes. In general, MCSs with 20–30 mmHg of compression are recommended for patients with varicose veins. Patients suffering from active ulcers usually tolerate up to 30–40 mmHg of compression while more resistant chronic venous insufficiency like lymphedema may require 40–50 mmHg or more [24]. A study showed that patients with symptoms of mild venous insufficiency benefit from wearing MCSs providing an ankle pressure of 10–20 mmHg. It was found that lower pressure is invalid while a higher pressure adds nothing [25]. This is true for occupational leg swelling but not for improving CVD [25,26].

Compression stockings are generally effective for four to six months and need to be replaced at the end of that time [3]. It’s best to put them on when subjects first get up and wear them constantly except when bathing and sleeping. 

#### 2.1.3. Application of Bandages

Bandages are usually utilized with high stretch or low stretch as well as single layer or multilayer. The compression pressure of less than 20 mmHg has been categorized as mild, 20–40 mmHg as medium, 40–60 mmHg as strong, and greater than 60 mmHg as very strong [27]. Compression bandages of 35–45 mmHg pressure at the ankle were proven in several studies to be safe and effective [27,28]. Novel bandage like Setopress^®^ make it easy to apply the right compression regardless of the limb shape or size. This is realized by the imprinting of a rectangle on the compressive material which, when stretched to a certain size, “looks like a square.” This provides the desired magnitude of compression (generally 30–35 mmHg) [3].

For the sake of subjects’ safety, there is obviously an upper limit of the compression pressure. For low stretch bandages, this upper limit is about 30 mmHg on the upper extremity while around 50–60 mmHg on the lower extremity [29]. In the upstanding position, a compression pressure exceeding 50 mmHg is required for a reduction of ambulatory venous hypertension and intermittent occlusion of incompetent veins during walking [30]. Such high intermittent interface pressure peaks exert a “massaging effect” and may be achieved by low stretch multilayer bandages than by elastic stockings [30,31].

### 2.2. Scar Management

A hypertrophic scar (HS) is a very typical skin complication resulting from dermal injury especially following severe burns. The scar partial is inevitably different from the other normal skin with regard to pigmentation, color, vascularity, thickness, and hardness, which may result in the self-abasement and stigmatization. Regarding physical comfort, the scar would lead to lots of symptoms including pruritus, pain, erythema, and more. Additionally, if the scar is close to a joint, articular stiffness may be caused by the scar contracture. Therefore, scars may lead to aesthetic, psychological, physiological, and functional problems to patients. Therefore, this inevitably undermines the quality of their life [32].

Compression garments have been utilized for the prophylaxis and treatment of the hypertrophic scar since the 1970s. It is also the first-line conservative management so far. However, the effectiveness of the pressure treatment is controversial and has little scientific evidence to support it. The mechanism of the pressure treatment is not clear enough from the current research. It is thought that possible mechanisms are reduced collagen formation and increased collagen lysis due to the reduction of blood flow and oxygen supply to the scar tissues and increased apoptosis [33,34].

To achieve the ideal result, the compression garment is recommended to be worn about 23 h per day and for 1 year until the scar matures. The propriate pressure is best kept at 20–30 mmHg [35,36] so the compression garments should be changed every 2 to 3 months to prevent a diminish in elasticity [37,38]. The defects of the compression garment are mainly in the aspects of limited use, depression pressure, and patient discomfort [39].

There are some areas which have a concave or flat shape that prevents pressure from being delivered by the pressure garment. In this area, the pressure garment alone cannot exert the required pressure. Therefore, it required additional padding or a face mask. There is a lot of research that had been conducted on developing the face mask for burn injury patients [40,41,42,43]. 

### 2.3. Orthopedic Supports

Knitted orthopedic supports are a kind of medical compression garments. Knitted orthopedic supports are commonly divided into three categories, according to preventive supports, functional supports, and post-operative/rehabilitative supports, and they could be utilized as knee braces, wrist braces, ankle braces, shoulder braces, elbow-braces, and calf, lumbar, and back supports [44]. Unfortunately, the compression requirements for orthopedic support are not standardized to date [45].

Orthopedic supports are generally customized for subjects based on a combination of the science of proprioception and targeted skeletal support technology. Appropriately designed orthopedic supports create a positive anatomical change in the body as well as an increase in body strength, enhance motor skills, and/or provide support to paraplegic patients, neonates, elderly, pregnant, and nursing women, and patients with motor disabilities [9]. 

Knitted orthopedic supports are usually manufactured with an anatomically-shaped knitted elastic fabric with some additional inserts like silicone, metallic, or other parts as a frame [46]. Orthopedic supports may also be comprised of other components for reinforcements or adjustable parts such as straps, fasteners, and hinges. All parts included support that can change the elasticity of the entire product.

### 2.4. Sportswear

Functional compression sportswear is supposed to help sportsmen enhance performance at the competition. Two principles should be primarily considered to guide the design and engineering of these sportswear. The first is applying compression on specific muscles to increase blood flow and the other is application of principles of an aerodynamics to reduce drag in high speed sports [2]. Depending on the requirements, the sportswear could be designed according to both principles or individually. In addition to functional features, aesthetics is also an important design criterion in this category.

Compression sportswear enhances performance in power-based activities mainly via several mechanisms. First, it is believed that augmented proprioception could be responsible for an improvement in the technique while reduced oscillatory displacement of muscles may promote enhanced neurotransmission and mechanics at the cellular and molecular level [8]. It boosts lactate removal, enhances oxygen supply, and improves recovery following exercise and training. Furthermore, the elasticity of the compression garment provides increased flexion and extension torque at the end range of extension and flexion, respectively. It may assist the hamstrings in controlling the leg at the end of the swing phase in sprinting [47]. Lastly, psychological factors are also perceived to improve performance [48,49].

Another viewpoint is that wearing compression sportswear is not helpful for enhancing sprint or throwing a performance, but could be beneficial to reduce post-exercise trauma, swelling, and perceived muscle soreness, and accelerate the recovery of force production [7,50,51,52,53]. Different conclusions on the effects of wearing compression sportswear may be related to the types of activities and sportswear and the individual differences. 

Research studies have conducted to discover the optimal effective and safe compression. With regard to swimsuits, compression below 7.5 mmHg exerted on a trunk was helpful for venous pump action as well as work efficiency. However, for shoulder and inguinal region, high compression would lead to a suppressed skin blood flow to peripheral parts and the delay of blood pressure recovery after exercise [54]. If the exerted pressure values of chest above 8.6 mmHg, the pressure values of waist exceeded 2.7 mmHg. If the pressure values of abdomen exceeded 4.2 mmHg, it will have an influence on the comfort of wearing swimsuits [55].

In addition, for the sportswear, the properties of chemical stability, UV resistance, air permeability, water vapor transmission, and washability should also be taken into consideration.

### 2.5. Body Shaping

Tight fitting garments are employed to beautify the body figure by compression on specific body parts to the required shapes [56]. The girdle is a typical type of pressure foundation wear worn by women to re-shape the lower part of the body by uplifting the hips and compressing the abdomen in order to enhance the aesthetic appearance of the wearer. According to the support capabilities, girdles could be classified as light, medium, and firm level of control. The firmer level of support is provided by tighter materials and more layers of fabrics. While shaping the women’s lower part of the body, it should not create discomfort nor any detrimental effects on wearer’s physiology [18].

The pressure exerted by girdles is a significant parameter since it is closely concerned with body shaping effects and wearing comfort. The optimum pressure of the ten different positions include the front tummy, left front tummy, right front tummy, left side, right side, left front lower, right front lower, left hips, right hips, and waist level are calculated. As the results reveal, most people can bear relatively higher pressure (about 11.5 mmHg) at the two sides and prefer lower pressure (about 4.5 mmHg) at the hip and the acceptable pressure for the waist is about 6.5 mmHg and the mean acceptable pressure is about 7.5 mmHg [57]. A study on subjects wearing girdles of various design, materials, patterns, and construction was conducted and found the discomfort pressure was above 30–40 mmHg [58]. Manufacturers can refer to the optimum clothing pressure distribution to develop and design girdles to satisfy the needs of most people.

The compression may not only relate to garment size and properties, but also to factors including body size and anatomy like human fat, resilience of muscle, and human bone structure. Furthermore, other subjective sensations such as tactile feeling, hot/wet feeling, and objective measures such as the change of body shape could be used in future work to evaluate the effectiveness of girdles.

### 2.6. Smart Compression Garments

Smart Compression Garments (SCG) are a kind of novel compression garments integrated with the pressure mapping system capable of assessing both muscular and skeletal motion and positioning [59,60]. It includes a wearable system, which could process information of soft tissue data of active muscle load, cruciate ligament forces, and *co*-contraction of paired muscles in real-time and is able to provide metrics for improved performance and safety [61]. 

Novel approaches in material-based sensor technology show that the integration of soft sensors within compression garments is possible. The Electromyography (EMG) and MEMS-based inertial sensors are also used in the SCG [59]. SCG is able to record, store, stream, and deliver muscle and joint data for real-time feedback to users and mainly function as a proprioceptive aid during recovery. By using more advanced and appropriate materials, sensors, and systems, future SCG will be expected to monitor the pressure distribution on the body and detect the vibration characteristics of muscles (soft tissues) and benefit for the subjects’ proprioceptive sensation, neuromuscular control, injury prevention, and performance enhancement [8].

The strong development challenge of current SCG is sensors-based activity recognition technology. For market product application, the transition of clinical accuracy into an affordable consumer-focused garment including the limitations of cost and reliability should be taken into account [62]. 

## 3. Fibers and Yarns

### 3.1. Types of Elastomeric Fibers and Yarns

The most prominent feature of the compression garment is its elastic mechanical properties that render it as an effective membrane stretching element acting on the curvilinear human body. To achieve this, elastic fibers and yarns that exhibit good extensibility and elastic recovery have been used to make the elastic fabrics including knitted fabrics. Elastomeric or spandex fibers and their continues filament yarns have been applied for this purpose including segmented polyurethane fibers, polyester ether fibers, polyester fibers like PBT poly (butylene terephthalate) (PBT) fiber and polytrimethylene terephthalate (PTT) fiber, olefin based elastomeric fibers, and bio-component fiber [63]. According to the extension, they can be classified into low elastic fiber with an elongation range from 20% to 150%, medium elastic fiber with elongation in the range of 150% to 390%, and the high elastic fiber with elongation up to 400% to 800%. Commercial synthetic elastic fibers used in compression garments normally have an extension break over 200% and exhibit rapid recovery when tension is released [63,64]. 

Lycra™ is the first popular commercial elastomeric fiber, which is invented and marketed by DoPont (E. I. du Pont de Nemours and Company, Wilmington, DE, USA) [65]. It is a synthetic linear macromolecule with a long chain consisting of at least 85% of segmented polyurethane along with the alternating hard and soft segments linked by urethane bonds [66]. The soft chain segments provide elasticity to fiber while the hard segments supply a molecular interaction force to fiber and guarantee the strength and stability of fiber [64]. More than 90% of the spandex fibers are produced with the method of solution dry spinning since this method supplies better heat resistance and tenacity than other methods like melt extrusion, reaction spinning, and solution wet spinning. 

Apart from elastomeric fibers, other fibers such as nylon 6 or nylon 66 filaments or cotton are employed to make elastic yarns. Covered yarn, core-spun yarn, and textured (or air-covered) yarn are the common elastic yarns utilized in the compression garments [67]. In a covered yarn, the elastomeric core yarn is wrapped by a covering filament yarn with *Z* or *S* twist. The covering can be done twice in double covered yarn while the second wrapping is completed in the opposite direction to achieve torque balance and stability. Core-spun yarn is achieved on a ring spinning machine where short staple fibers like cotton are around an elastomeric core yarn. The textured yarn consists of elastomeric yarn and false-twisted covering yarn. It is made by a combined process of false-twist texturing and air-jet mingling. Among the three kinds of yarns, the covered and core-spun elastomeric yarns are for high extension applications and textured yarns are for low to medium stretch requirements [66]. 

### 3.2. Characteristics of Elastomeric Yarns

The mechanical properties including tensile properties and elastic recovery properties are the most important for the performance of the products contained elastomeric yarns. In addition to this, elastomeric fibers should also satisfy the requirements for forming a finished product such as stability under dyeing and finishing conditions, easy processing with non-elastic yarns, and stability under normal washing conditions [64,68]. 

There are usually two types for the elastomeric fibers inserted in the fabrics. One kind is bare threads inserted together with other yarns or through interlacing and the other type is covered or core-spun elastomeric yarns. For the blending yarns, the tensile properties are influenced by material composition, processing, and process parameters.

**Figure 1:**
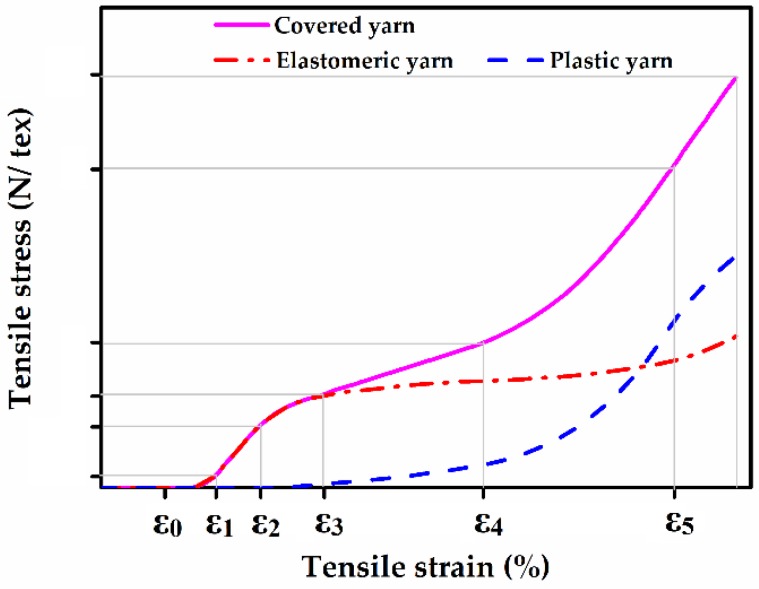
Stress-strain curves of a covered yarn with elastic core and covering yarns. Images adapted and reproduced with permission from Reference [69]. Copyright SAGE Publications 2012.

An analysis based on the elastomeric yarn wrapped with nylon will explain elongation performance. Figure 1 shows the theory of the superposition of two yarn models including elastomeric and plastic yarns in the elongation dynamics and they all contribute to the general model with the continuous purple curve [69]. From the beginning to ε_0_, the strain is mainly acquired from the elastomeric yarn with high elastic modulus, as shown by the red dashed curve. While the contribution of the polyamide yarn with high plastic properties, which is shown by the blue dotted curve, occurs progressively and between a given range of elongation ε_0_ and ε_3_. With specific machine settings, the properties of the elastomeric yarn become dominant at an extreme degree of elongation. The global constitutive law of a material is contributed by the constitutive law of all the components consisting of the material.

**Figure 2:**
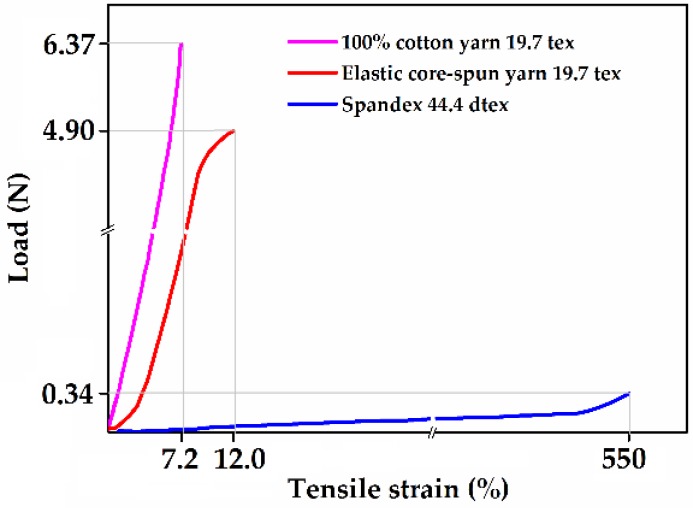
Load-elongation curves of the 100% cotton yarn, the spandex, and the elastic core-spun yarn. Images adapted and reproduced with permission from Reference [70]. Copyright SAGE Publications 2004.

The mechanical properties of elastomeric core-spun yarns were significantly affected by spandex and the outer yarns. Figure 2 shows the load-elongation curve of the 100% cotton yarn (19.7 tex), the spandex (44.4 dtex/4f), and the elastomeric core-spun yarn (19.7 tex) [70]. It was found that the elastomeric part of core-spun yarns such as spandex did not contribute much to yarn strength while a non-elastic part of core-spun vortex yarns restricted the stretch limit of core-spun yarns [71]. 

In terms of compression garments, the suitable and stable pressure is the most crucial. For sportswear or body shaping garments, the low or medium elastomeric fibers could meet the elastic requirements while, for a medical purpose, high elastomeric fibers should be used to obtain the desired pressure. Within the usage range of the tensile curve, the tensile strain is expected to increase slowly when the tensile stress rose. Therefore, the pressure would be stabilized in a relative range. This also means there is a wide range of applications for the compression garments to satisfy different sizes and movements. 

## 4. Fabric Construction and Mechanical Properties

### 4.1. Knitting Construction and Fabrication

**Figure 3:**
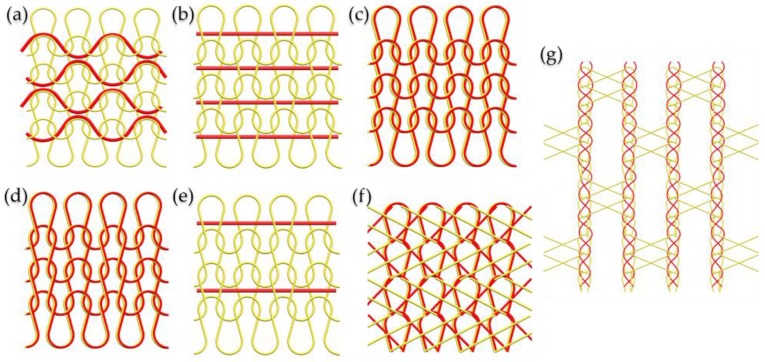
Typical knitting construction for elastic fabrics (red line is elastic yarn and yellow line is ground yarn): (**a**) Elastic yarns are as laid-in stitch in jersey. (**b**) Elastic yarns are as weft inlay stitch in jersey. (**c**) Elastic yarns are as plating stitch in jersey. (**d**) Elastic yarns are as plating stitch in rib, (**e**) Elastic yarns are as weft inlay stitch in jersey. (**f**) Elastic yarns are knitted on the back bar with 1–0/1–2// tricot stitch. (**g**) Elastic yarns are as laid-in stitch to produce power-net construction.

Since elastic knitted fabrics take the highest proportion of the compression garments, the knitting construction is reviewed then. The weft knitting and warp knitting are the two categories of the knitting technology. For the weft knitting, the flat knitting machine and circular knitting machine consists of a single jersey, interlock, a double jersey, and rib machines, which are always used. In terms of warp knitting, Tricot, Raschel, and a double-needle-bar Reschel machine are employed. The details of the construction and knitting methods are listed in Table 1. Figure 3 shows the typical knitting constructions utilized in the fabrication of elastic fabrics.

On these knitting machines, various elastic fabrics could be fabricated and used to make compression garments. They are usually knitted with at least two types of yarn including a ground yarn to ensure thickness and stiffness of knitted fabric and an inlay-yarn to generate compression. The elastomeric inlay-yarn can be inlayed, floated, or plated into a knitted structure. Higher levels of compression are mainly achieved by increasing thickness of the elastic core of the inlay-yarn even though adjustments may also be made to the knitting process including the knitting construction and elastic yarn insertion density [45,72,73]. 

According to the application of different kinds of compression garments, one should select the appropriate material, knitting construction, and method to achieve the elastic characteristic. 

### 4.2. Fabric Mechanical Properties

Compression garments are made of elastic fabric. Extensibility and elastic recovery are its most important characteristics because of which compression garments are able to exert continuous pressure on the human body. During the wearing of compression garments, the hysteresis of fabric and dynamic elastic properties are also the significant factors influencing the actual compression.

Research studies have been done to prove that elastomeric yarns and knitting construction all influence the elasticity of the fabric. A study conducted by Senthilkumar et al. [74] found that, at different extension levels, spandex-plaited cotton fabric has better dynamic elastic recovery than spandex core cotton spun fabric. In another study, Cooper et al. [75] tested the stretch and recovery properties of different stretch fabrics with all-cotton, nylon, and polyester/spandex core yarns and indicated that yarn type and inter fiber friction may play a significant part in the stretch and recovery properties. Research studies also indicated that elastic recovery depends on the compression force provided, the length of time that the force is applied for, and the length of time that the fabric is allowed to recover [2].

Apart from extensibility and elastic recovery, hysteresis is also an important phenomenon for elastic fabrics. Hysteresis reflects the stress relaxation of elastic fabric when it has been subjected to repeated stretching and recovery [76]. The hysteresis phenomenon was first discovered by Ng [77]. He found that elastic fabrics had relaxed their stress significantly under stretching and the degree of stress relaxation would increase with prolonged time under stretching. Studies also found that fabrics containing elastomeric yarns had severe hysteresis problems under constant deformation. In addition, stress relaxation would result in pressure degradation [78].

In the compression garments, stiffness is also a significant mechanical property of fabric that affects the compression performance. It is defined as the change of compression exerted by a garment when the girth is increased or decreased and includes static stiffness and dynamic stiffness. Dynamic stiffness is important because it play a vital role in the actual use. Stiffness is related to the elasticity of the material and the construction of the fabric [79]. The research showed that there is a positive correlation between the static and dynamic stiffness indices, but the dynamic stiffness has a slightly higher value [76]. In clinical practice, the compression garment should be selected when considering the prospective stiffness [80].

Since clothing pressure largely depended on its biaxial extension and stress relaxation properties [81], research studies have been conducted to find the relationship between the clothing pressure of knitted fabrics and stress under extension and recovery processes [82,83]. The pressure behavior of tubular knitted elastic fabrics at different extension levels during 48 hours was investigated and it was found that the stitch length and extension rate are the vital factors that affects the interfacial pressure and pressure reduction [84]. The effects of spandex feeding rate and the fabric structure of elastic fabric were also analyzed [85]. Most of the compression garments have to be worn about 23 hours per day, which means the fabric corresponded under stretching at a given extension level for a long and continuous time before tension was released [86]. Practical applications also verify the opinion that the pressure of elastic fabric is time-dependent. As a result, slackening occurs in compression garments when patients wear them over a prolonged period of time and pressure decay affects the effectiveness of compression therapy [87]. 

Under conditions of wear, garments should adapt to human movement by relatively sliding and partially stretching between garments and the human body [85]. A study indicated that the pressure performance of a compression garment is closely related to multi-mechanical behavior of its knitted fabrics such as stretching, shearing, bending, and fabric surface friction [88]. 

## 5. Garment Design and Evaluation System

### 5.1. Garment Design

Normal compression garments are produced in a regular cut-and-sew method. First, flat knitted elastic fabrics are knitted to the correct size and shape (fully fashioned) and, in a subsequent step, to sew them together [89]. Most of sportswear with low compression are produced with this method. Although it has the advantages of lower cost and production flexibility, the disadvantages are more obvious since the seams always cause skin irritation and easy to make breaks on the garments.

One-piece construction, also known as the whole garment, is commonly utilized in the compression garments design. Double needle bar warp machine, flat knit machine, and circular knit machine have their advantages for knitting whole seamless compression garments with minimal or no cutting and sewing processes [90]. The seamless technology is often used for high compression garments like medical compression stocking, swimwear, and support garments. 

Different parts of compression garments may be designed since various knitting construction methods like single jersey, mesh, or rib are used to achieve the required fabric properties and corresponding compression. For instance, the construction of the seamless knitting pattern could be designed to apply the appropriate pressure to the specific muscles. For compression sportswear, the mesh structure would be used into the underarms under the chest areas and at the center of the back to acquire body fitting and better air permeability in some cases [91]. 

As commercial products with various garment design and construction, not all compression garments are properly fit to achieve the optimal effects. The excessive improper compression could enlarge the unpleasant and uncomfortable feeling during the wearing of the compression garment and even restrict blood supply and cause debilitation. Therefore, customized compression garments for a variety of specific functions are becoming more prevalent [51]. 

### 5.2. Design System

Various novel technologies like body scanning, the three-dimensional (3D) garment model, and pattern generation are beneficial to customized garment manufacturing area as well as the customized compression garment development. 

To develop a design system for customized compression garment, three important steps should always be considered. The first step is to acquire body shape and size. This step is primarily for achieving the exact measurement of the human body. The second stage is to investigate a mathematical model to determine the size and properties of compression garment and the pressure exerted. The last stage is to develop a computerized system to design the compression garment based on the mathematical model and to verify the system. 

Usually, 3D digital scanning is used to get body information such as curvature of body parts. A 3D compression garment model is generated by a mathematical model based on the fabric mechanical properties with desired pressures exerted on body and body curvature [92,93,94,95]. Then the 3D garment model was flattened into a two-dimensional (2D) pattern design to construct the compression garment [96]. Because the 2D pattern corresponds to a fabricated 3D shape compression garment to realize the desired pressure, the relationship between the 3D mesh surface and a 2D planar pattern needs to be investigated. A physical/geometric approach to model was conducted by Wang [56]. The 2D meshes were computed and can generate a specific pressure when folded into the 3D body. 

Most compression garment design systems collect the fabric mechanical properties and the curvature radius of body parts to build a 3D compression garment model. Therefore, it is beneficial for achieving the desired compression from specific elastic fabrics. While, for the whole design engineering process, all the critical factors included in the design process should be taken into account and added into the design system. Therefore, the tensile properties of yarns, the construction types, and physical properties of fabrics show an effect on the physical properties of the final garments and applicable usage types and so need to be added in the design system [44]. 

Although the mechanical properties of body parts also have a great influence on the pressure distribution, the fleshy parts sustain pressure better than bony parts. The present design system of the compression garment doesn’t take human anatomy into consideration [97].

### 5.3. Evaluation for Compression Garments

Generally, to ensure high treatment efficacy and health safety, application of the appropriate pressure is the most important consideration. In addition, thermo-moisture comfort, tactile conform, toxicity, and sanity properties should be taken into account.

Three essential components including the garment fit, the garment slip, and fabric stretch are important to meet the skin strain requirements [98]. The garment fit is determined by the ratio of garment size to body size and the nature of garment design, which provides the space allowance for skin strain. For compression garments, the garment size is always designed smaller than body size and the reduced proportion is the reduction factor. The garment slip is usually determined by the coefficient of friction between skin and fabric and between each layer of garments. Since compression garments are normally closely fitted with a pretension, the “fabric stretch” determines the pressure exerted on the body parts. If a garment has high friction and stretching resistance, high clothing pressure is likely to be exerted on the body, which could result in discomfort sensations. The critical strain areas of the body are the knee, the seat, the back, and the elbows [99].

Compression garments are generally to be worn by patients continuously for at least several hours and sometimes during intense exercise. Therefore, it is of the utmost importance to take the comfort characteristics of such garments into consideration. In addition, design incorporates features such as human anatomy, ergonomics, material choice, final application, and service life as well as cost to be evaluated when choosing products [100]. Furthermore, side-effects should not be ignored since an inappropriate compression garment will affect the energy, work efficiency, and health of the wearer. 

## 6. Pressure Measurement and Modeling

### 6.1. Pressure Measurement

The pressure of compression garments consists of static pressure and dynamic pressure and the measurement methods include in vitro and in vivo. Basically, the in vitro pressure is calculated from the force-extension curve of the elastic fabric combined with Laplace’s Law [101]. The measurement in vivo is always conducted with various interface pressure devices.

#### 6.1.1. Pressure Sensors

The early techniques utilized to measure pressure are with hysteresis, low sensor accuracy, and poor conformity to body curvature [102] such as electro-pneumatic [103] and fluid-filled pressure transducers [104]. By applying piezoresistive elements such as strain gauges and force-sensing resistors, resistive pressure devices were proven to have higher accuracy [105]. However, the piezoresistive sensors were found sensitive to temperature and the sensor sensitivity is limited at forces under 10 mmHg. 

The capacitive pressure sensor was also developed to measure the pressure by translating signals of pressure changes into electrical capacitance variation. This kind of pressure transducer showed not only higher sensitivity and flexibility, lower temperature dependency, and also lower power consumption than piezoresistive devices [106]. It was generally used on rehabilitation engineering prosthesis and orthosis with high interface pressure [107]. A commercially capacitive sensor, the Pliance X System (Germany-Novel Electronics, Munich, Germany) has been developed and applied for static interface pressure measurement between skin and compression garments [108].

#### 6.1.2. Static Pressure Measurement

An ideal interface pressure device should usually meet the requirements of sensitivity, small and thin in dimensions, highly flexible, and the measurement range as low as 0–50 mmHg [109]. It should be capable to display a continuous output not affected by temperature or moisture. Above all, for pressure measurement between body and garments, the sensor should be highly conformed to the body contour and free from error measurement on curved surfaces. 

Current devices for measuring garment static pressure are principally used in the medical field such as the Kikuhime^®^ pressure monitor, SIGaT tester^®^, and PicoPress^®^ pressure monitor [76,110,111,112]. They satisfy the requirements for static pressure measurement. Nevertheless, these devices except PicoPress^®^ pressure monitor are not well suited to dynamic pressure measurement due to limited portability, communication, and capacity during specific sports conditions [50,112,113].

#### 6.1.3. Dynamic Pressure Measurement

During activity and exercise, as muscles and tendons exert force for motion and stability, the limb size of the body experiences great changes [114]. That means the pressure between the body and garments is dynamic. It is significant to detect the dynamic pressure for the compression garment design to achieve better physiological efficacy. 

For the commercial products, the PicoPress^®^ transducer, which is a kind of pneumatic pressure transducer, could be used as a dynamic pressure tracing in vivo by connecting with a software program and may be left under compression for several days [112]. The measured value will be higher than the actual value because the direct interface measurement affects the radius of curvature [115].

However, there is little research on the dynamic pressure measurement. A study developed a wearable wireless pressure monitoring device, which consisted of six 18mm diameter low profile pressure sensors placed on different positions of the body such as calf, thigh, and buttocks regions, and could measure pressures in the range of 5–50 mmHg [114]. The system was reported to have an acceptable accuracy and precision to measure the dynamic pressure when running and had the advantages for portability and memory. For practical applications, the disadvantage for unstable measurements may appear because the sensors are attached on the body. They have a potential to shift during strenuous exercise. The preferable but more challenging way is inserted or integrated sensors and circuit in compression garments improve practicability and acceptability.

### 6.2. Pressure Modeling

Direct measurement of the garment pressure on the human body has several limitations and is impractical for routine tests. Therefore, it is expected to predict the garment pressure by numerical simulation in the early stages of the design process.

As far as research is concerned, there are mainly three methods known as Laplace’s Law, the finite element method, and the volumetric subdivision scheme, which are used for pressure modeling to predict the compression garments’ pressure.

Many scholars have combined the body curvature radius with the biaxial extension properties of elastic fabrics based on the Laplace’s Law to predict the clothing pressure on the human body [86,93,98,116]. This method is a basic and convenient tool to calculate the rough pressure, but it doesn’t take the elastic modulus of the human body and body motion into consideration. Therefore, the pressure calculated doesn’t conform to the real condition. 

The finite element method is commonly used and 3D models of human body are developed for numerical simulation of dynamic garment pressure during wear. Research conducted by Zhang et al. [117] presented a mechanical model for numerical simulation of 3D dynamic contact pressure using a finite element method based on the theory of contact mechanics. It is verified that the model can simulate the compression garment pressure during wearing with a reasonable accuracy. While the model assumed the body is rigid and the friction between the body and garment was neglected. A more accurate finite element model was developed from the reconstruction of geometrical shapes of the commercial 3D anatomic male skin and the skeleton model [118]. It showed a big local pressure variation along the cross section because of the influence from the curvature of the human body and the anatomic bony and muscular structure. Another numerical simulation based on finite element analysis was studied by Liu et al. [119,120]. In the study, the 3D bio-mechanical mathematical model was developed through pressure objective testing, material physical estimation, the 3D geometry finite element female leg model, and numerical simulation. The simulated model could be used for numerically simulating the compression stocking’s special deformations, the surface pressure magnitude, distribution in longitudinal directions, and dynamic mechanical interactions between the human leg and stocking during wear. The model has consistently shown a reasonable agreement with the experimental measurements. However, the differences on the practical wearing state, the limitation of the pressure sensor, and the definition of biomaterials caused the error especially at the ankle part.

Alternatively, Wang employed a geometric interpolatory volumetric subdivision scheme over the hexahedron lattice to simulate the elastic human body deformation and the pressure distribution when wearing tight-fitting clothing [121]. In this simulation method, the elastic human body model and fabric mass-spring model were used. Through volumetric subdivision procedures, a sequence of lattices converging to a continuous and deformed region are obtained, which indicates that the displacement of any vertex in the recursively refined human body could be achieved. 

The modeling methods are convenient in application while the more accurate model needs to be developed because most of the body modeling is based on over-simplified geometrical assumptions and may not match well with the actual anthropometric dimensions and physical properties [122]. 

## 7. Conclusions and Future Outlook

In this article, we conducted a comprehensive review on compression garments for medical therapy and sportswear. As a major part of the review, the garments design including the material and its properties, knitting construction and methods, and the design system has been carefully studied and discussed. Afterward, special attention has been raised on the pressure measurement and modeling of compression garments. Furthermore, the areas of compression garments utilized in chronic venous disease and scar management, orthopedic supports, sportswear, and body shaping have introduced the action principle and appropriate pressure for different effects and body parts.

By choosing proper materials, construction and garment design according to the body curvature and anatomy and the suitable pressure for body parts, the pressure therapy and sportswear could acquire optimal effects. As the stiffness index of the compression garment is important in the practice clinic, it is independent of the compression class and it is necessary to refine the current classification system for the compression garment especially the dynamic stiffness index. For example, it is more effective to prevent edema with higher stiffness, so the compression stocking with a high stiffness could be prescribed.

The new compression garments are expected to be designed to “remember” and keep their shape. Novel materials and construction are helpful for the development of compression garments. For instance, shape memory materials could be used in the specific part of the compression stocking to keep the consistent effective compression. Alternatively, several systems of elastic yarns with various elasticity could be combined in one fabric but with different constructions in the same course. Therefore, there is a possibility that the low elasticity yarn and high elasticity yarn play a role at different stages during the process. Despite for providing the required amount of compression therapy, the novel compression garments are expected to be more sheer and comfortable. 

The biggest challenge for the research of compression garments is the pressure prediction and variation during the wearing. For the better wearing instruction of compression garments, the pressure criteria for specific body parts, real time pressure display, and overpressure warning are the research direction.

## Figures and Tables

**Table 1:** Knitting construction and fabrication methods.

Knitting Method	Knitting Machine	Knitting Construction	Isotropy in Deformation	Applications
Weft knitting	Single jersey	Elastic yarns are knitted or inlaid or plating to knit jersey or double jersey, fleece, pique	Elastic deformation in the wale and course direction	Sportswear; Medical compression garments
	Double jersey			
	Interlock	Bare or Covered elastic yarns are inlaid or plating	Elastic deformation in the wale direction	
	Rib			
	Flat knitting	1. covered elastic yarns are knitted or inlaid or plating 2. space fabric	Elastic deformation in the wale and course direction	
Warp knitting	Tricot	Elastic yarns are knitted on the back bar with 1–0/1–2// tricot stitch to knit fabrics with plain jersey and mesh	Elastic deformation in the wale and course direction	Swimwear; tight-fitting garments
	Raschel	Elastic yarns are as laid-in stitch to produce power-net construction	Elastic deformation in the course direction	Swimwear; tight-fitting garments; support garments
	Double-needle-bar Raschel	1. Elastic yarns are in the surface bar to knit space fabric 2. Circular elastic fabric	Elastic deformation mainly in the course direction	

## References

[1] MacRae B.A., Cotter J.D., Laing R.M. (2011). Compression garments and exercise: Garment considerations, physiology and performance. Sports Med..

[2] Wang L., Felder M., Cai J.Y. (2011). Study of properties of medical compression garment fabrics. J. Fiber Bioeng. Inform..

[3] Felty C.L., Rooke T.W. (2005). Compression therapy for chronic venous insufficiency. Semin. Vasc. Surg..

[4] Martin H.A. (1878). The india-rubber bandage for ulcers and other diseases of the legs. Br. Med. J..

[5] Staley M.J., Richard R.L. (1997). Use of pressure to treat hypertrophic burn scars. Adv. Wound Care.

[6] Born D.P., Sperlich B., Holmberg H.C. (2013). Bringing light into the dark: Effects of compression clothing on performance and recovery. Int. J. Sport Physiol..

[7] Duffield R., Portus M. (2007). Comparison of three types of full-body compression garments on throwing and repeat-sprint performance in cricket players. Br. J. Sport Med..

[8] Fu W., Liu Y., Fang Y. (2013). Research advancements in humanoid compression garments in sports. Int. J. Adv. Robot. Syst..

[9] Gupta D. (2011). Functional clothing—Definition and classification. Indian J. Fibre Text. Res..

[10] Denton M. (1972). Fit, stretch and comfort. Textiles.

[11] Tanaka S., Midorikawa T., Tokura H. (2006). Effects of pressure exerted on the skin by elastic cord on the core temperature, body weight loss and salivary secretion rate at 35 °C. Eur. J. Appl. Physiol..

[12] Eberhardt R.T., Raffetto J.D. (2014). Chronic venous insufficiency. Circulation.

[13] Robertson L., Evans C., Fowkes F. (2008). Epidemiology of chronic venous disease. Phlebology.

[14] Meissner M.H., Gloviczki P., Bergan J., Kistner R.L., Morrison N., Pannier F., Pappas P.J., Rabe E., Raju S., Villavicencio J.L. (2007). Primary chronic venous disorders. J. Vasc. Surg..

[15] Jawien A. (2003). The influence of environmental factors in chronic venous insufficiency. Angiology.

[16] Nicolaides A., Allegra C., Bergan J., Bradbury A., Cairols M., Carpentier P., Comerota A., Delis C., Eklof B., Fassiadis N. (2008). Management of chronic venous disorders of the lower limbs guidelines according to scientific evidence. Int. Angiol..

[17] Fletcher J., Moffatt C., Partsch H., Vowden K., Vowden P. (2013). Principles of Compression in Venous Disease. A Practitioner’s Guide to Treatment and Prevention of Venous Leg Ulcers.

[18] Fan C.-M. (2005). Venous pathophysiology. Semin. Interven. Radiol..

[19] Bachelor E.P. (2001). Varicose veins and telangiectasias: Diagnosis and treatment. Plast. Reconstr. Surg..

[20] Schuren J., Mohr K. (2010). Pascal’s law and the dynamics of compression therapy: A study on healthy volunteers. Int. Angiol..

[21] Li Y., Dai D.X. (2006). Biomechanical engineering of compression stockings. Biomechanical Engineering of Textiles and Clothing.

[22] Sugisawa R., Unno N., Saito T., Yamamoto N., Inuzuka K., Tanaka H., Sano M., Katahashi K., Uranaka H., Marumo T. (2016). Effects of compression stockings on elevation of leg lymph pumping pressure and improvement of quality of life in healthy female volunteers: A randomized controlled trial. Lymphat. Res. Biol..

[23] Wollina U., Abdel-Naser M.B., Mani R. (2006). A review of the microcirculation in skin in patients with chronic venous insufficiency: The problem and the evidence available for therapeutic options. Int. J. Low. Exterm. Wounds.

[24] Hill J., Howatson G., van Someren K., Davidson S., Pedlar C. (2014). Pressures exerted by commercially available lower limb compression garments. Br. J. Sports Med..

[25] Amsler F., Blättler W. (2008). Compression therapy for occupational leg symptoms and chronic venous disorders—A meta-analysis of randomised controlled trials. Eur. J. Vasc. Endovasc..

[26] Partsch H., Winiger J., Lun B. (2004). Compression stockings reduce occupational leg swelling. Dermatol. Surg..

[27] Partsch H., Clark M., Mosti G., Steinlechner E., Schuren J., Abel M., Benigni J.P., Coleridge-Smith P., Cornu-Thénard A., Flour M. (2008). Classification of compression bandages: Practical aspects. Dermatol. Surg..

[28] Weingarten M.S. (2001). State-of-the-art treatment of chronic venous disease. Clin. Infect. Dis..

[29] Partsch H., Damstra R., Mosti G. (2011). Dose finding for an optimal compression pressure to reduce chronic edema of the extremities. Int. Angiol..

[30] Partsch B., Partsch H. (2005). Calf compression pressure required to achieve venous closure from supine to standing positions. J. Vasc. Surg..

[31] Partsch H. (2012). Compression therapy: Clinical and experimental evidence. Ann. Vasc. Dis..

[32] Widgerow A.D., Chait L.A. (2011). Scar management practice and science: A comprehensive approach to controlling scar tissue and avoiding hypertrophic scarring. Adv. Skin Wound Care.

[33] Gauglitz G.G., Korting H.C., Pavicic T., Ruzicka T., Jeschke M.G. (2011). Hypertrophic scarring and keloids: Pathomechanisms and current and emerging treatment strategies. Mol. Med..

[34] Reno F., Sabbatini M., Lombardi F., Stella M., Pezzuto C., Magliacani G., Cannas M. (2003). In vitro mechanical compression induces apoptosis and regulates cytokines release in hypertrophic scars. Wound Repair Regen..

[35] Partsch H., Mortimer P. (2015). Compression for leg wounds. Br. J. Dermatol..

[36] Van den Kerckhove E., Stappaerts K., Fieuws S., Laperre J., Massage P., Flour M., Boeckx W. (2005). The assessment of erythema and thickness on burn related scars during pressure garment therapy as a preventive measure for hypertrophic scarring. Burns.

[37] Zurada J.M., Kriegel D., Davis I.C. (2006). Topical treatments for hypertrophic scars. J. Am. Acad. Dermatol..

[38] Sharp P.A., Pan B., Yakuboff K.P., Rothchild D. (2016). Development of a best evidence statement for the use of pressure therapy for management of hypertrophic scarring. J. Burn Care Res..

[39] Johnson J., Greenspan B., Gorga D., Nagler W., Goodwin C. (1994). Compliance with pressure garment use in burn rehabilitation. J. Burn Care Rehabil..

[40] Parry I., Hanley C., Niszczak J., Sen S., Palmieri T., Greenhalgh D. (2013). Harnessing the transparent face orthosis for facial scar management: A comparison of methods. Burns.

[41] Rogers B., Chapman T., Rettele J., Gatica J., Darm T., Beebe M., Dilworth D., Walsh N. (2003). Computerized manufacturing of transparent face masks for the treatment of facial scarring. J. Burn Care Rehabil..

[42] Van den Kerckhove E., Stappaerts K., Boeckx W., Van den Hof B., Monstrey S., Van der Kelen A., De Cubber J. (2001). Silicones in the rehabilitation of burns: A review and overview. Burns.

[43] Yip C., Mehmood Z., Shah M. (2008). Lego^®^ as a customisable pressure garment insert. Burns.

[44] LáZáR K. Application of knitted fabrics in technical and medical textiles. Proceedings of the 45th International Congress (IFKT).

[45] Ališauskienė D., Mikučioniené D., Milašiute L. (2013). Influence of inlay-yarn properties and insertion density on the compression properties of knitted orthopaedic supports. Fibres Text. East. Eur..

[46] Mikučionienė D., Milašiūtė L. (2017). Influence of knitted orthopaedic support construction on compression generated by the support. J. Ind. Text..

[47] Doan B.K., Kwon Y.H., Newton R.U., Shim J., Popper E.M., Rogers R.A., Bolt L.R., Robertson M., Kraemer W.J. (2003). Evaluation of a lower-body compression garment. J. Sport Sci..

[48] Higgins T., Naughton G.A., Burgess D. (2009). Effects of wearing compression garments on physiological and performance measures in a simulated game-specific circuit for netball. J. Sci. Med. Sport.

[49] Pearce A.J., Kidgell D.J., Grikepelis L.A., Carlson J.S. (2009). Wearing a sports compression garment on the performance of visuomotor tracking following eccentric exercise: A pilot study. J. Sci. Med. Sport.

[50] Scanlan A.T., Dascombe B.J., Reaburn P.R., Osborne M. (2008). The effects of wearing lower-body compression garments during endurance cycling. Int. J. Sport Physiol..

[51] Kraemer W.J., Flanagan S.D., Comstock B.A., Fragala M.S., Earp J.E., Dunn-Lewis C., Ho J.-Y., Thomas G.A., Solomon-Hill G., Penwell Z.R. (2010). Effects of a whole body compression garment on markers of recovery after a heavy resistance workout in men and women. J. Strength Cond. Res..

[52] Kraemer W.J., Bush J.A., Wickham R.B., Denegar C.R., Gomez A.L., Gotshalk L.A., Duncan N.D., Volek J.S., Newton R.U., Putukian M. (2001). Continuous compression as an effective therapeutic intervention in treating eccentric-exercise-induced muscle soreness. J. Sport Rehabil..

[53] Sperlich B., Haegele M., Achtzehn S., Linville J., Holmberg H.C., Mester J. (2010). Different types of compression clothing do not increase sub-maximal and maximal endurance performance in well-trained athletes. J. Sport Sci..

[54] Morooka H., Nakahashi M., Morooka H., Kitamura K. (2001). Effects of clothing pressure exerted on a trunk on heart rate, blood pressure, skin blood flow and respiratory function. J. Text. Mach. Soc. Jpn..

[55] Xu D.-F., Liu D.-Y., Wu Z.-M. (2012). Analysis of physiological response by pressure developed by female swimsuit. J. Beijing Inst. Cloth. Technol..

[56] Wang C.C., Tang K. (2010). Pattern computation for compression garment by a physical/geometric approach. Comput. Aided Des..

[57] Chan A., Fan J. (2002). Effect of clothing pressure on the tightness sensation of girdles. Int. J. Cloth. Sci. Technol..

[58] Makabe H., Momota H., Mitsuno T., Ueda K. (1991). A study of clothing pressure developed by the girdle. J. Jpn. Res. Assoc. Text. End-Uses.

[59] Belbasis A., Fuss F.K., Sidhu J. (2015). Muscle activity analysis with a smart compression garment. Procedia Eng..

[60] Belbasis A., Fuss F.K. (2015). Development of next-generation compression apparel. Procedia Technol..

[61] Belbasis A., Fuss F.K., Sidhu J. (2015). Estimation of cruciate ligament forces via smart compression garments. Procedia Eng..

[62] Chen L., Hoey J., Nugent C.D., Cook D.J., Yu Z. (2012). Sensor-based activity recognition. IEEE Trans. Syst. Man Cybern. Part C Appl. Rev..

[63] Hu J., Lu J., Zhu Y. (2008). New developments in elastic fibers. Polym. Rev..

[64] Senthilkumar M., Anbumani N., Hayavadana J. (2011). Elastane fabrics—A tool for stretch applications in sports. Indian J. Fibre Text. Res..

[65] Brown R.P. (2001). Polymers in Sport and Leisure.

[66] Hu J., Lu J. (2015). Recent developments in elastic fibers and yarns for sportswear. Textiles for Sportswear.

[67] Bhat G., Chand S., Yakopson S. (2001). Thermal properties of elastic fibers. Thermochim. Acta.

[68] Kumar B., Das A., Alagirusamy R. (2014). Effect of material and structure of compression bandage on interface pressure variation over time. Phlebology.

[69] Bruniaux P., Crepin D., Lun B. (2012). Modeling the mechanics of a medical compression stocking through its components behavior: Part 1—Modeling at the yarn scale. Text. Res. J..

[70] Su C.-I., Maa M.-C., Yang H.-Y. (2004). Structure and performance of elastic core-spun yarn. Text. Res. J..

[71] Gazi Ortlek H., Ulku S. (2007). Effects of spandex and yarn counts on the properties of elastic core-spun yarns produced on murata vortex spinner. Text. Res. J..

[72] Krimmel G. (2009). The construction and classification of compression garments. Template for Practice: Compression Hosiery in Upper Body Lymphoedema.

[73] Ališaukienė D., Mikučionienė D. (2012). Investigation on alteration of compression of knitted orthopaedic supports during exploitation. Mater. Sci..

[74] Senthilkumar M., Anbumani N. (2011). Dynamics of elastic knitted fabrics for sports wear. J. Ind. Text..

[75] Cooper A., Robinson H.M., Reeves W.A., Sloan W.G. (1965). Mechanism for stretch and recovery properties of certain stretch fabrics1. Text. Res. J..

[76] Van Der Wegen-franken K., Tank B., Neumann M. (2008). Correlation between the static and dynamic stiffness indices of medical elastic compression stockings. Dermatol. Surg..

[77] Sau-fun Ng Yip F. (1994). Medical clothing: The stress relaxation and shrinkage of pressure garments. Int. J. Cloth. Sci. Technol..

[78] Šajn D., Geršak J., Flajs R. (2006). Prediction of stress relaxation of fabrics with increased elasticity. Text. Res. J..

[79] Hirai M., Koyama A., Miyazaki K., Iwata H., Kominami Y. (2012). Interface pressure and stiffness in different combinations of compression material. Phlebology.

[80] Hirai M., Niimi K., Miyazaki K., Iwata H., Sugimoto I., Ishibashi H., Ota T., Kominami Y. (2011). Development of a device to determine the stiffness of elastic garments and bandages. Phlebology.

[81] Ito N., Inoue M., Nakanishi M., Niwa M. (1995). The relation among the biaxial extension properties of girdle cloths and wearing comfort and clothing pressure of girdles. J. Jpn. Res. Assoc. Text. End-Uses.

[82] Hui C., Ng S. (2003). Theoretical analysis of tension and pressure decay of a tubular elastic fabric. Text. Res. J..

[83] Yamada T., Matsuo M. (2009). Clothing pressure of knitted fabrics estimated in relation to tensile load under extension and recovery processes by simultaneous measurements. Text. Res. J..

[84] Maleki H., Aghajani M., Sadeghi A., Jeddi A.A.A. (2011). On the pressure behavior of tubular weft knitted fabrics constructed from textured polyester yarns. J. Eng. Fabr. Fibers.

[85] Wang Y.R., Zhang P.H., Zhang Y.P. (2014). Experimental investigation the dynamic pressure attenuation of elastic fabric for compression garment. Text. Res. J..

[86] Macintyre L., Ferguson R. (2013). Pressure garment design tool to monitor exerted pressures. Burns.

[87] Geršak J., Šajn D., Bukošek V. (2005). A study of the relaxation phenomena in the fabrics containing elastane yarns. Int. J. Cloth. Sci. Technol..

[88] Liu R., Kwok Y.-L., Li Y., Lao T.-T. (2010). Fabric mechanical-surface properties of compression hosiery and their effects on skin pressure magnitudes when worn. Fibres Text. East. Eur..

[89] Mermelstein S., Hale D. (2001). New developments in the manufacture of circular knitting machines for the production of medical textiles. Medical Textiles.

[90] Kanakaraj P., Ramachandran R. (2010). Seamless garment: Needle selection techniques and applications. Pak. Text. J..

[91] Troynikov O., Watson C. (2015). Knitting technology for seamless sportswear. Textiles for Sportswear.

[92] Salleh M.N.B., Acar M., Burns N.D. (2011). Customised pressure garment development by using 3d scanned body image. Res. J. Text. Appar..

[93] Macintyre L., Baird M., Weedall P. (2004). The study of pressure delivery for hypertrophic scar treatment. Int. J. Cloth. Sci. Technol..

[94] Maklewska E., Nawrocki A., Ledwoń J., Kowalski K. (2006). Modelling and designing of knitted products used in compressive therapy. Fibres Text. East. Eur..

[95] Yıldız N. (2007). A novel technique to determine pressure in pressure garments for hypertrophic burn scars and comfort properties. Burns.

[96] Salleh M.N.B., Lazim H.B.M., Othman S.N.B., Merican A.F.M.B.A. (2013). Development of a flexible customised compression garment pattern design system. Int. J. Adv. Mech. Syst..

[97] Gupta D. (2011). Design and engineering of functional clothing. Indian J. Fibre Text. Res..

[98] Kirk W., Ibrahim S. (1966). Fundamental relationship of fabric extensibility to anthropometric requirements and garment performance. Text. Res. J..

[99] Choudhury A.R., Majumdar P., Datta C. (2011). Factors affecting comfort: Human physiology and the role of clothing. Improving Comfort in Clothing.

[100] Pereira S., Anand S., Rajendran S., Wood C. (2007). A study of the structure and properties of novel fabrics for knee braces. J. Ind. Text..

[101] Partsch H., Partsch B., Braun W. (2006). Interface pressure and stiffness of ready made compression stockings: Comparison of in vivo and in vitro measurements. J. Vasc. Surg..

[102] Ferguson-Pell M., Hagisawa S., Bain D. (2000). Evaluation of a sensor for low interface pressure applications. Med. Eng. Phys..

[103] Partsch H. (2005). The static stiffness index: A simple method to assess the elastic property of compression material in vivo. Dermatol. Surg..

[104] Ferguson-Pell M., Cardi M.D. (1993). Prototype development and comparative evaluation of wheelchair pressure mapping system. Assist Technol..

[105] Maklewska E., Nawrocki A., Kowalski K., Andrzejewska E., Kuzański W. (2007). New measuring device for estimating the pressure under compression garments. Int. J. Cloth. Sci. Technol..

[106] Chavan A.V., Wise K.D. (2001). Batch-processed vacuum-sealed capacitive pressure sensors. J. Microelectromech. Syst..

[107] Hafner J., Lüthi W., Hänssle H., Kammerlander G., Burg G. (2000). Instruction of compression therapy by means of interface pressure measurement. Dermatol. Surg..

[108] Lai C.H., Li-Tsang C.W. (2009). Validation of the pliance x system in measuring interface pressure generated by pressure garment. Burns.

[109] Swain I. (2005). The measurement of interface pressure. Pressure Ulcer Research.

[110] Partsch H., Clark M., Bassez S., Benigni J.P., Becker F., Blazek V., Caprini J., Cornu-Thénard A., Hafner J., Flour M. (2006). Measurement of lower leg compression in vivo: Recommendations for the performance of measurements of interface pressure and stiffness. Dermatol. Surg..

[111] Stolk R., Wegen van der-Franken C., Neumann H. (2004). A method for measuring the dynamic behavior of medical compression hosiery during walking. Dermatol. Surg..

[112] Partsch H., Mosti G. (2010). Comparison of three portable instruments to measure compression pressure. Int. Angiol..

[113] Trenell M.I., Rooney K.B., Sue C.M., Thomspon C.H. (2006). Compression garments and recovery from eccentric exercise: A 31P-MRS study. J. Sports Sci. Med..

[114] McLaren J., Helmer R., Horne S., Blanchonette I. (2010). Preliminary development of a wearable device for dynamic pressure measurement in garments. Procedia Eng..

[115] Shahidi A.M., Dias T., Nandasiri G.K. (2018). The impact of direct and indirect pressure measuring systems on the pressure mapping for the medical compression garments. World Acad. Sci. Eng. Technol. Int. J. Fash. Text. Eng..

[116] Macintyre L., Baird M. (2006). Pressure garments for use in the treatment of hypertrophic scars—A review of the problems associated with their use. Burns.

[117] Zhang X., Yeung K., Li Y. (2002). Numerical simulation of 3d dynamic garment pressure. Text. Res. J..

[118] Lin Y., Choi K.-F., Luximon A., Yao L., Hu J., Li Y. (2011). Finite element modeling of male leg and sportswear: Contact pressure and clothing deformation. Text. Res. J..

[119] Liu R., Kwok Y.-L., Li Y., Lao T.-T., Dai X.Q., Zhang X. (2007). Numerical simulation of internal stress profiles and three-dimensional deformations of lower extremity beneath medical graduated compression stocking (GCS). Fiber Polym..

[120] Liu R., Kwok Y.-L., Li Y., Lao T.-T., Zhang X., Dai X.Q. (2006). A three-dimensional biomechanical model for numerical simulation of dynamic pressure functional performances of graduated compression stocking (GCS). Fiber Polym..

[121] Wang J.-M., Luo X.-N., Li Y., Dai X.-Q., You F. (2005). The application of the volumetric subdivision scheme in the simulation of elastic human body deformation and garment pressure. Text. Res. J..

[122] Wang Y., Cui Y., Zhang P., Feng X., Shen J., Xiong Q. (2011). A smart mannequin system for the pressure performance evaluation of compression garments. Text. Res. J..

